# Supplementary material for: Rapid and Simple Detection of Burkholderia gladioli in Food Matrices Using RPA-CRISPR/Cas12a Method
Source: Foods. 2023 Apr 24;12(9):1760. doi: 10.3390/foods12091760 (PMC10178126; doi:10.3390/foods12091760)
Supplement: Supplementary file 1 [file foods-12-01760-s001.zip › foods-2350946-supplementary.pdf]

# Supplementary Material

**Table S1** Nine sets of primer sequences and sizes.(All the primers were designed based on the gene fragment of number EF552059 in genebank）

| Primers | | Sequence of primers | Length of product/bp |
|---|---|---|---|
| R1 | Forward | 5'-GTCTTTGTCATTGGCGATTGAGCCAGTCAA-3' | 144bp |
| | Reverse | 5'-ATACAATCACAACCCGGATAGTTTTCACGC-3' | |
| R2 | Forward | 5'-GCACCGTCTTGATAAGGCGGGGGTCGTTGG-3' | 231bp |
| | Reverse | 5'-CTGACTGGCTCAATCGCCAATGACAAAGAC-3' | |
| R3 | Forward | 5'-CTTTGTCATTGGCGATTGAGCCAGTCAAAGGATA-3' | 142bp |
| | Reverse | 5'-ATACAATCACAACCCGGATAGTTTTCACGCCCAT-3' | |
| R4 | Forward | 5'-CTTTGTCATTGGCGATTGAGCCAGTCAAAGGATA-3' | 139bp |
| | Reverse | 5'-CAATCACAACCCGGATAGTTTTCACGCCCATCTC-3' | |
| R5 | Forward | 5'-CCGTCTTGATAAGGCGGGGGTCGTTGGTTCGAAT-3' | 224bp |
| | Reverse | 5'-CTGGCTCAATCGCCAATGACAAAGACTCGAGTCA-3' | |
| R6 | Forward | 5'-CTTGATAAGGCGGGGGTCGTTGGTTCGAATCC-3' | 210bp |
| | Reverse | 5'-CGCCAATGACAAAGACTCGAGTCAACTGACCC-3' | |
| R7 | Forward | 5'-ATAAGGCGGGGGTCGTTGGTTCGAATCCAACC-3' | 206bp |
| | Reverse | 5'-CGCCAATGACAAAGACTCGAGTCAACTGACCC-3' | |
| R8 | Forward | 5'-CGGGGGTCGTTGGTTCGAATCCAACCAGACCC-3' | 200bp |
| | Reverse | 5'-CGCCAATGACAAAGACTCGAGTCAACTGACCC-3' | |

| | | | |
|---|---|---|---|
| R9 | Forward | 5'-CCGTCTTGATAAGGCGGGGGTCGTTGGTTCGAAT-3' | 214bp |
| | Reverse | 5'-CGCCAATGACAAAGACTCGAGTCAACTGACCC-3' | |

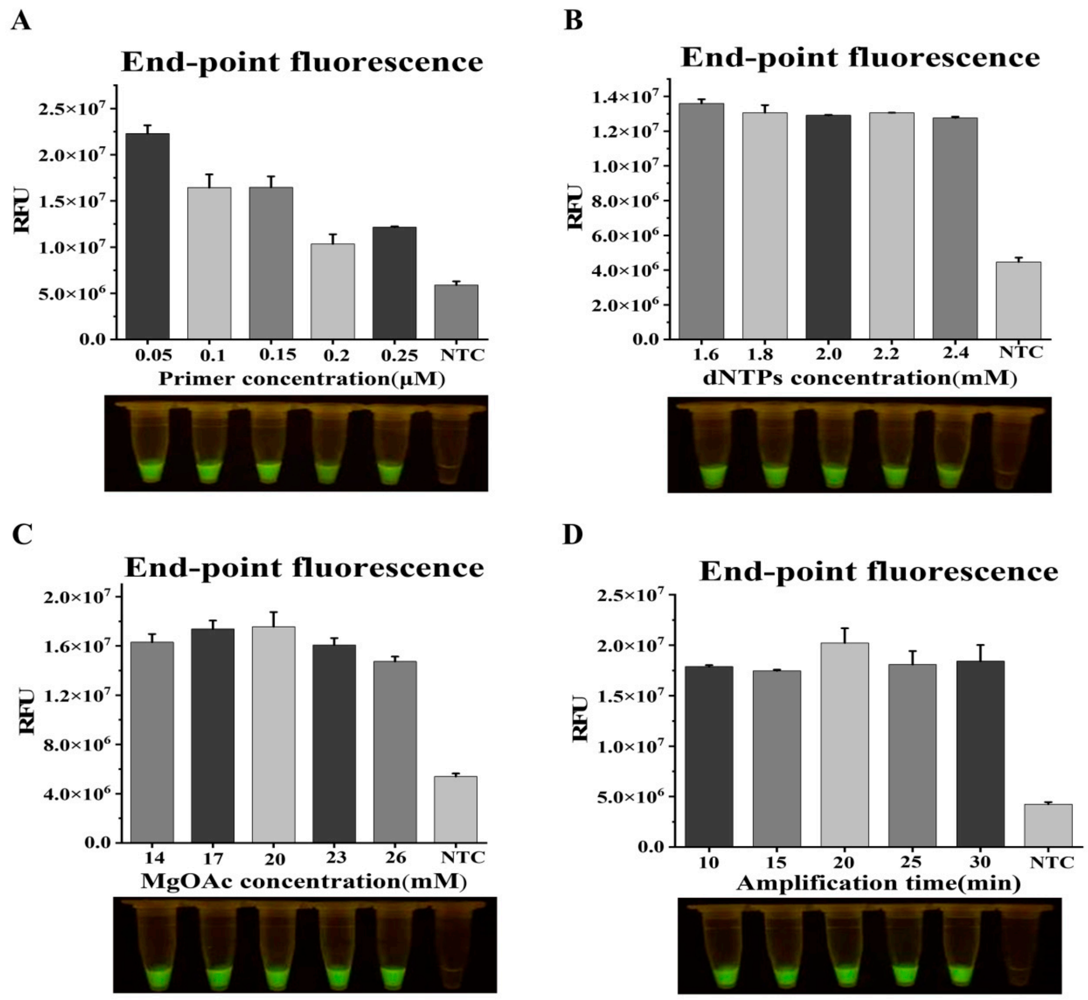


Figure S1. Optimization of RPA amplification system. (A) Primer concentration. (B) dNTPs concentration. (C) MgOAc concentration. (D) Amplification time. NTC: nontarget control.

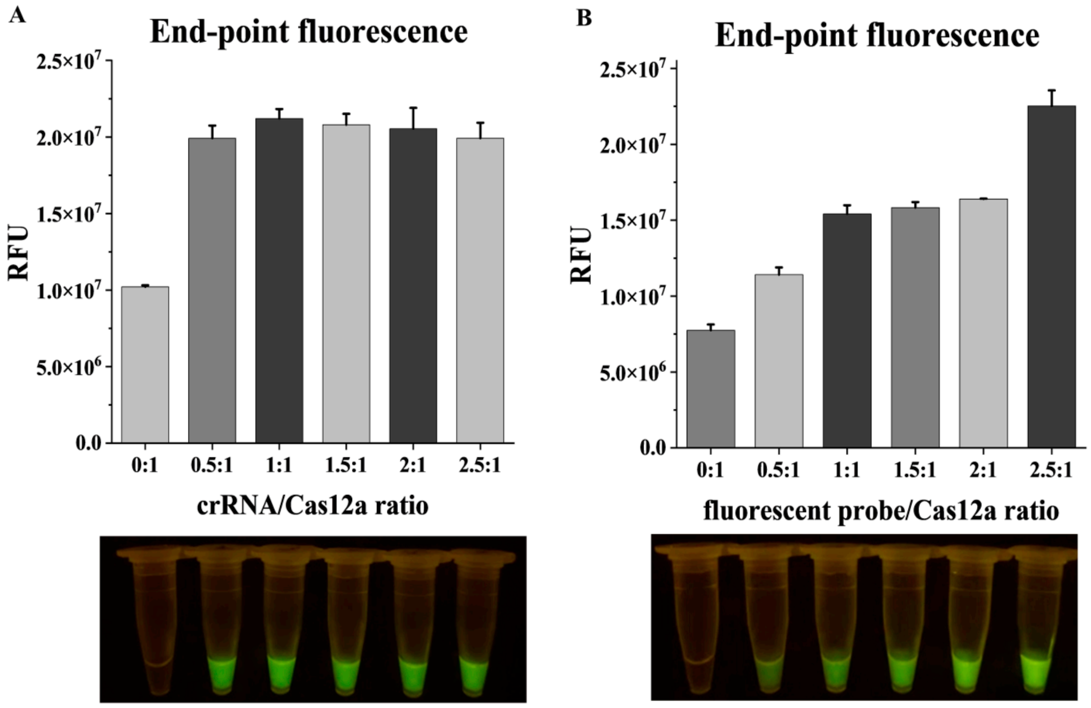


Figure S2. Optimization of Cas system.(A) Fluorescence generated from different crRNA/Cas12a ratios. (B) Fluorescence generated from different FQ-probe/Cas12a ratios. NTC: nontarget control.
